# A machine learning cardiac magnetic resonance approach to extract disease features and automate pulmonary arterial hypertension diagnosis

**DOI:** 10.1093/ehjci/jeaa001

**Published:** 2020-01-30

**Authors:** Andrew J Swift, Haiping Lu, Johanna Uthoff, Pankaj Garg, Marcella Cogliano, Jonathan Taylor, Peter Metherall, Shuo Zhou, Christopher S Johns, Samer Alabed, Robin A Condliffe, Allan Lawrie, Jim M Wild, David G Kiely

**Affiliations:** 1 Department of Infection, Immunity and Cardiovascular Disease, University of Sheffield, Western Bank, Sheffield S10 2TN, UK; 2 INSIGNEO, Institute for In Silico Medicine, The University of Sheffield, The Pam Liversidge Building, Sir Frederick Mappin Building, F Floor, Mappin Street, Sheffield, S1 3JD, UK; 3 Department of Computer Science, The University of Sheffield, 211 Portobello, Sheffield, S1 4DP, UK; 4 Radiology Department, Sheffield Teaching Hospitals NHS Foundation Trust, Glossop Rd, Sheffield S10 2JF, UK; 5 Sheffield Pulmonary Vascular Disease Unit, Royal Hallamshire Hospital, Sheffield Teaching Hospitals NHS Foundation Trust, Glossop Rd, Sheffield S10 2JF, UK

**Keywords:** machine learning, tensor, pulmonary arterial hypertension, diagnosis, right ventricle

## Abstract

**Aims:**

Pulmonary arterial hypertension (PAH) is a progressive condition with high mortality. Quantitative cardiovascular magnetic resonance (CMR) imaging metrics in PAH target individual cardiac structures and have diagnostic and prognostic utility but are challenging to acquire. The primary aim of this study was to develop and test a tensor-based machine learning approach to holistically identify diagnostic features in PAH using CMR, and secondarily, visualize and interpret key discriminative features associated with PAH.

**Methods and results:**

Consecutive treatment naive patients with PAH or no evidence of pulmonary hypertension (PH), undergoing CMR and right heart catheterization within 48 h, were identified from the ASPIRE registry. A tensor-based machine learning approach, multilinear subspace learning, was developed and the diagnostic accuracy of this approach was compared with standard CMR measurements. Two hundred and twenty patients were identified: 150 with PAH and 70 with no PH. The diagnostic accuracy of the approach was high as assessed by area under the curve at receiver operating characteristic analysis (*P* < 0.001): 0.92 for PAH, slightly higher than standard CMR metrics. Moreover, establishing the diagnosis using the approach was less time-consuming, being achieved within 10 s. Learnt features were visualized in feature maps with correspondence to cardiac phases, confirming known and also identifying potentially new diagnostic features in PAH.

**Conclusion:**

A tensor-based machine learning approach has been developed and applied to CMR. High diagnostic accuracy has been shown for PAH diagnosis and new learnt features were visualized with diagnostic potential.

## Introduction

Pulmonary arterial hypertension (PAH) is a life-shortening condition, and if untreated, it leads to right heart failure and death with a median survival of less than 3 years. The condition has an insidious onset with disease usually advanced at diagnosis.^1^ In response to distal vascular remodelling, the proximal pulmonary vasculature becomes dilated and stiffened.^2–5^ As the disease progresses the right ventricular (RV) dilates, hypertrophies, and deteriorates.^6–8^ In addition, other features including changes in interventricular septal configuration,^9^^,^^10^ atrial size and function,^11–13^ and pericardial and pleural effusions can occur.^14^^,^^15^ Echocardiography is recommended in suspected pulmonary hypertension (PH) via assessing systolic pulmonary arterial pressure.^16^^,^^17^

Cardiovascular magnetic resonance (CMR) imaging protocols can assess a large number of characteristics in a single examination. In contrast to the 10’s of millions of voxels within an imaging dataset, derived data used for diagnostic and prognostic purposes is limited. Image analysis is time-consuming, particularly in PAH where the automatic segmentation algorithms typically fail due to ventricular deformation and reproducibility is lower for the right ventricle than the left.

Volumetric assessment of the right ventricle with CMR is superior to that achieved with echocardiography,^18^ though remains challenging.^19^ Machine learning can rapidly and consistently identify relevant information from vast 3D/4D data usually inaccessible to the reporting physician. Multilinear subspace learning^20^ is an emerging tensor-based machine learning approach that reduces the dimensionality of multidimensional data by directly mapping their tensor representations to a low-dimensional space, with recent application to automatically identify features in registered brain magnetic resonance imaging images to discriminate between different brain conditions.^21^ However, this multilinear subspace learning approach to extract, visualize, and interpret diagnostic features have not previously been applied to cardiovascular imaging. Success of such a machine learning diagnostic approach could improve standardization and diagnostic rates, particularly in less experienced centres. Even in experienced centres, machine learning may allow a more focused, enhanced, and standardized assessment by considering computer learnt diagnostic features alongside expert interpretation of the images.

To our knowledge, no studies have used multilinear subspace learning to extract, interpret, and visualize the range of diagnostic features in suspected PH, not to mention without the necessity for manual segmentation.

The primary aim of this study was to develop and test multilinear subspace learning for CMR imaging to identify and learn diagnostic features in patients with suspected PAH without the need for manual segmentation. The secondary aim was to visualize and interpret key discriminative features identified by the machine learning method.

## Methods

### Patients

Consecutive treatment naive patients with PAH or no evidence of PH (no PH) referred for CMR between December 2014 and February 2017 at a PH referral centre were identified. Diagnosis was made following multidisciplinary team assessment. Inclusion required CMR and right heart catheterization to be performed within 48 h. Ethical approval was granted from the local ethics committee and institutional review board for this retrospective study and written consent was waived (ref c06/Q2308/8).

### MR acquisition

CMR was performed on a GE HDx (GE Healthcare, Milwaukee, USA) whole-body scanner at 1.5 T using an eight-channel cardiac coil. Short-axis cine images were acquired using a cardiac gated multislice balanced SSFP sequence (20 frames per cardiac cycle, slice thickness 8 mm, FOV 48, matrix 512 × 512, BW 125 KHz/pixel, TR/TE 3.7/1.6 ms). Mid-chamber cine images in addition to a stack of images in the short-axis plane with slice thickness of 8 mm (2 mm interslice gap) were acquired fully covering both ventricles from base to apex, end-systole was considered as the smallest cavity area, and end-diastole was defined as the first cine cardiac phase of the R-wave triggered acquisition or largest volume. Patients were scanned in the supine position with a surface coil and retrospective electrocardiogram gating.

### Image preprocessing

#### Registration

CMR images from different patients/sequences were registered using paired landmark points. Three landmarks were manually labelled on the first frame of each image sequence and reviewed (AJS). The short-axis image landmarks are inferior and superior hinge points and the inferolateral inflection point of the RV free wall. The four-chamber images landmarks are left ventricular (LV) apex, mitral, and tricuspid annuli. One image sequence in a dataset was considered the reference sequence to register the rest of the sequences against. A geometric transformation was fit to these three landmark point pairs, including translation, rotation, scaling, and reflection (flipping). This transformation was then used to warp a particular sequence against the reference sequence to accomplish registration. The distances between the warped landmarks and the reference landmarks were computed and the maximum among the three distance values was examined for quality assurance.

#### Masking

We studied two elliptical masks (small and large) to focus the machine learning approach on the relevant anatomy, i.e. a region including the whole heart. The two masks were automatically generated from five boundary landmarks manually drawn (A.J.S.) on the first frame of the reference sequence, which needs to be done only once for short axis or four chambers.

#### Scaling

To reduce the negative effect (e.g. overfitting) of the small training sample size (∼200) on the machine learning model, we rescaled the original spatial resolution of 512 × 512 (0.94 mm) to smaller scales 32 × 32 (15 mm), 64 × 64 (7.50 mm), 128 × 128 (3.75 mm), and 256 × 256 (1.89 mm) to find a resolution best for prediction.

### Machine learning methodology

Machine learning builds models to automatically learn and improve from data examples rather than being explicitly programmed. The machine learning system in this article consists of (i) a feature extraction step to learn a low-dimensional representation from high-dimensional input, (ii) a feature selection step to focus on a small portion of extracted features, and (iii) a classifier to learn a decision function from selected features that distinguish which candidate category a new observation belongs. Due to the small training sample size (∼200) relative to the high input dimensionality (32 × 32 × 20 = 20 480 even at the smallest scale 32 × 32), complex machine learning models such as random forests and neural networks are susceptible to overfitting. Therefore, we choose simple linear methods in the following, which are also more transparent and interpretable than complex, non-linear methods. Alternative choices at each step are possible but not the focus of this article.

**Figure 1:**
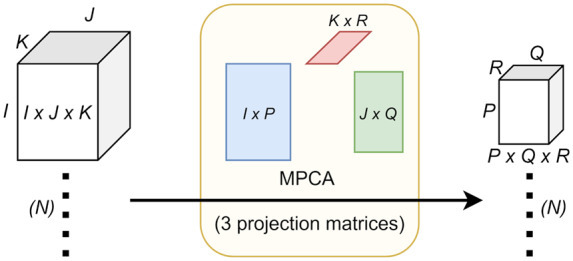
Illustration of multilinear principal component analysis (MPCA). PCA is a traditional linear dimensionality reduction method that extracts low-dimensional features from high-dimensional input. MPCA extends PCA to tensor representations of data. In this article, the input to MPCA is *N* samples of CMR sequences with size *I* × *J* × *K*, with a spatial dimension of *I* × *J* and a time dimension of *K* (frames). MPCA maps each *I* × *J* × *K* tensor to a low-dimensional *P* × *Q* × *R* tensor (*P < I*, *Q < J*, and *R < K*) using three projection matrices of size *I* × *P*, *J* × *Q*, and *K* × *R*. During training, these three matrices are optimized to maximize the variation captured in the *N* mapped *P* × *Q* × *R* tensors and these optimized matrices are the output of the MPCA learning algorithm. During testing, the learnt three matrices map a new *I* × *J* × *K* tensor input of *I* × *J* × *K* into a *P* × *Q* × *R* tensor as its low-dimensional representation.

**Figure 2:**
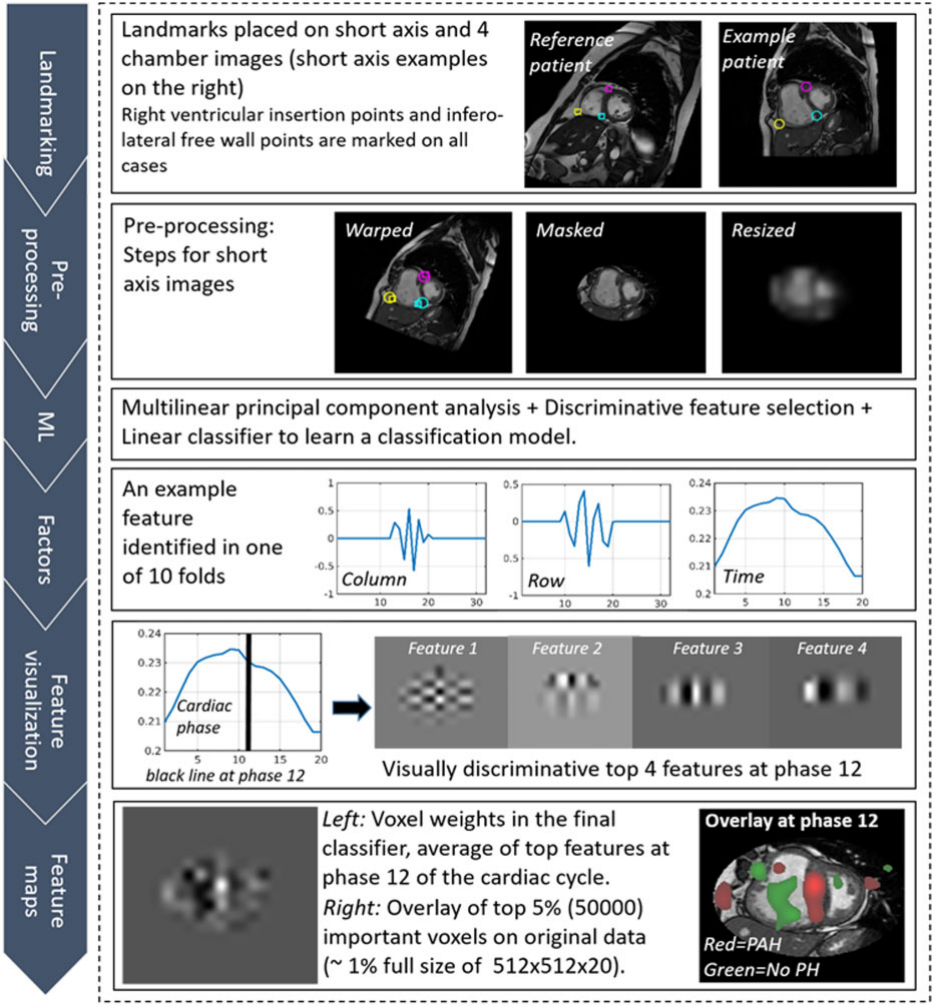
Proposed machine learning workflow: manual landmarking, pre-processing steps with registration, masking, and rescaling, machine learning steps, visualization of learnt factors and features, and feature maps. The learnt factors in the fourth row are highly interpretable: the column (size *I* × *1*) and row (size *J* × *1*) factors capture spatial variations closely resemble wavelets popular in representing fundamental patterns in natural images, and the time factor (size *K* × *1*) shows the cardiac phase.

A sequence of *K* CMR images of a standard size *I × J* is viewed as a single sample (example) of data, with size *I × J × K*, which is a multidimensional array called a *tensor* in mathematics. There were *N* training sequences available for training. We first used multilinear principal component analysis (MPCA), a fundamental multilinear subspace learning algorithm that extends principal component analysis (PCA) to tensor input, to reduce the dimensionality of each (tensor) sample from *I × J × K* to MPCA features of size *P × Q × R* typically with *P < I*, *Q < J*, and *R < K* using three projection matrices of size *I × P*, *J × Q*, and *K × R*^22^ (see *Figure 1*). Each MPCA feature corresponds to an eigentensor that is a rank-one tensor with (*I + J + K*) parameters, much less than the number of parameters (*I × J × K*) for an eigenvector associated with each feature if PCA is used, greatly reducing overfitting.^20^ To further reduce the dimensionality and improve interpretability, we used Fisher’s discriminant ratio to sort the *P × Q × R* features in a descending order and selected only the first *S* most discriminative features from these *P × Q × R* features. The hyperparameter *S* is determined by 10-fold cross-validation on the training data (i.e. inner cross-validation) using one of the two linear classifiers, support vector machine (SVM) with a linear kernel and logistic regression (LR), which are more efficient, more interpretable, and less prone to overfitting. Finally, we fed these *S* selected features from all the training data to the same linear classifier (SVM or LR) to learn a final classification model. *Figure 2* is a flow diagram that illustrates the machine learning process with example learnt features. In particular, the fourth row of *Figure 2* shows that the learnt factors are highly interpretable, where the column (size *I × 1*) and row (size *J × 1*) factors closely resemble wavelets and the time factor (size *K × 1*) shows the cardiac phase. The proposed multilinear subspace learning approach was tested on a computer with four cores at 2.10 GHz and 32 GB memory. Not including the time for landmarking, the diagnosis process takes less than 1 s for one CMR image sequence. In total, the whole approach takes <10 s, where the landmarking time dominates.

### Cardiac volume mass and function image analysis

Cardiac volume, mass, and function analysis were prospectively acquired on a GE Advantage Workstation 4.1 (GE Healthcare, Milwaukee, USA). The observer, a CMR radiographer with 10 years CMR experience was blinded to the patient clinical information, cardiac catheter parameters, and machine learning data. Right and left endocardial and epicardial surfaces were traced manually from the stack of short-axis cine images to obtain RV end-diastolic (RVEDV) and end-systolic (RVESV), stroke volume (RVSV) and ejection fraction (RVEF), LV end-diastolic (LVEDV) and end-systolic volumes (LVESV), stroke volume (LVSV) and ejection fraction (LVEF), RV end-diastolic mass (RV mass), and LV end-diastolic mass (LV mass). For calculation of LV mass, the interventricular septum was considered as part of the left ventricle. Ventricular mass index (VMI) was defined as RV mass divided by LV mass, as previously described.^23^^,^^24^

### Right heart catheterization and clinical assessment

Diagnostic classification of PAH was made using standard criteria following multi-professional assessment. Right heart catheterization was performed using a balloon-tipped 7.5-Fr thermodilution catheter (Becton-Dickinson, USA). Right heart catheterization was performed via the internal jugular vein using a Swan-Ganz catheter. Right heart catheterization indices required to define PAH were mean pulmonary artery pressure (mPAP) ≥25 mmHg at rest with a pulmonary arterial wedge pressure (PAWP) of ≤15 mmHg. Pulmonary vascular resistance (PVR) was determined as follows: PVR = (mPAP − PAWP)/cardiac output (CO). CO was measured by thermodilution technique. Cardiac index is calculated by CO/body surface area. No PH was defined as suspected PH with mean pulmonary arterial pressure at right heart catheterization less than 25 mmHg.

### Statistical analysis

Comparisons of CMR between patients with PAH and patients without PH were analysed using an independent *t*-test for continuous data, and χ^2^ and Fisher’s exact test for categorical data. Receiver operating characteristic (ROC) analysis was used to test the diagnostic strength of established CMR indices and multilinear subspace learning for the detection of the presence or absence of PAH. ROC curve analysis results are presented as area under the curve (AUC). Equal sensitivity and specificity were chosen from the ROC to report the accuracy (=sensitivity=specificity), positive predictive value (PPV), and negative predictive values (NPVs).

Ten-fold cross-validation was used to evaluate the proposed machine learning approach in terms of AUC. The patients were divided into 10 disjoint subsets (folds). We used nine subsets for training (learning the MPCA projection, determine *S*, and learning the classifier) and the remaining one subset for prediction (evaluation). This process was repeated 10 times so that each subset was used for prediction exactly once, with the average over 10 repeats reported. In addition, a subgroup analysis was performed assessing the accuracy for differentiation of patients with and without idiopathic PAH (IPAH).

A *P*-value <0.05 was determined to be statistically significant. The CMR indices were analysed using SPSS 22 (SPSS, Chicago, IL, USA) to obtain the statistics. The proposed machine learning approach was implemented and studied using MATLAB (version R2017b, MathWorks). The core MATLAB code is available at http://www.mathworks.com/matlabcentral/fileexchange/26168.

## Results

### Patients

**Figure 3:**
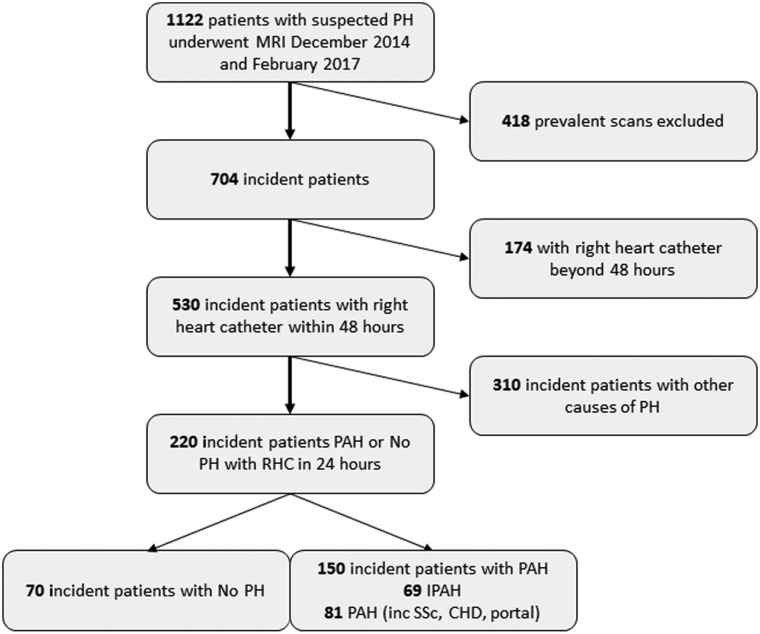
Patient flow diagram.

Of 1122 patients with suspected PH at their diagnostic visit, 220 patients with PAH or no PH were identified having CMR and RHC within 48 h (see *Figure 3*). One hundred and fifty patients were identified with PAH and 70 patients were found not to have PH. Among 150 patients with PAH, 69 patients were diagnosed with IPAH, 58 with PAH associated with connective tissue disease, 11 with PAH associated with portal hypertension, 10 with congenital heart disease, and 2 patients with PAH associated with drugs and toxins. *Table 1* presents the demographics, right heart catheter, and CMR indices for patients with and without PAH, and IPAH alone. Patients with PAH had higher mRAP, mPAP, PVR, and lower cardiac index and mixed venous oxygen saturations, all with *P* < 0.0001 compared to patients with no PH. In addition, higher RV end-diastolic volume and end-systolic volume and lower RV ejection fraction (all *P* < 0.001) were recorded for patients with PAH vs. patients without PH.

**Table 1:** Demographics, diagnostic, right heart catheter, and magnetic resonance imaging data

	No PH (*n* = 70)	PAH (*n* = 150)	*P*-value	IPAH (*n* = 69)	*P*-value
Demographics					
Age (years)	61 (17)	64 (13)	0.26	64 (13)	0.25
Sex female (%)	73	70	0.75	61	0.15
WHO FC I/II/III/IV	I (1), II (31), III (36), IV (2)	II (7), III (126), IV (17)		II (1), III (55), IV (13)	
Diagnosis					
IPAH		69			
CTD		58			
Portal		11			
CHD		10			
Drugs and toxins		2			
Right heart catheter					
mRAP (mmHg)	6 (3)	11 (6)	<0.0001	12 (6)	<0.0001
sPAP (mmHg)	33 (5)	74 (23)	<0.0001	85 (17)	<0.0001
dPAP (mmHg)	11 (3)	28 (10)	<0.0001	32 (9)	<0.0001
mPAP (mmHg)	20 (3)	46 (14)	<0.0001	53 (10)	<0.0001
PAWP (mmHg)	10 (3)	11 (3)	0.023	11 (3)	0.03
CI (L/min/m^2^)	3.0 (0.8)	2.5 (0.8)	<0.0001	2.2 (0.8)	<0.0001
PVR (dyns)	158 (67)	750 (450)	<0.0001	917 (429)	<0.0001
SvO_2_ (%)	71.1 (5.8)	64.5 (9.8)	<0.0001	60.1 (8.4)	<0.0001
CMR					
RVEDVi (mL/m^2^)	68 (21)	93 (38)	<0.0001	105 (38)	<0.0001
RVESVi (mL/m^2^)	32 (12)	60 (33)	<0.0001	72 (35)	<0.0001
RVEF (%)	53 (9)	38 (13)	<0.0001	33 (12)	<0.0001
RVSVi (mL/m^2^)	35 (13)	33 (14)	0.32	33 (12)	0.27
VMi (%)	0.26 (0.15)	0.47 (0.21)	<0.0001	0.57 (0.20)	<0.0001
Septal angle (degrees)	139 (10)	170 (23)	<0.0001	182 (19)	<0.0001

CI, cardiac index; CTD, connective tissue disease; dPAP, diastolic pulmonary arterial pressure; IPAH, idiopathic pulmonary arterial hypertension; mPAP, mean pulmonary arterial pressure; mRAP, mean right atrial pressure; PAH, pulmonary arterial hypertension; PAWP, pulmonary arterial wedge pressure; PVR, pulmonary vascular resistance; RVEDVi, right ventricular end-diastolic volume index; RVEF, right ventricular ejection fraction; RVESVi, right ventricular end-systolic volume index; RVSVi, right ventricular stroke volume index; sPAP, systolic pulmonary arterial pressure; SvO_2_, mixed venous oxygen saturations; VMI, ventricular mass index; WHO, World health organization.

### Learnt features, interpretation, and discovery

The average number of learnt features identified following MPCA and feature selection with 10-fold cross-validation on four-chamber images were 22 (out of 1215 total features on average, after MPCA) and 49 (out of 1242 total features on average) for no PH vs. PAH and IPAH, respectively. More features were identified on short-axis images though the total numbers of features were smaller, 66 (out of 212 total features on average) and 92 (out of 252 total features on average) for differentiating no PH vs. PAH and IPAH, respectively. These numbers were determined in a data-driven approach on the training data. Because this machine learning model is fully linear, these learnt features can be mapped back to the original images to determine voxel-wise weights obtained from linear classifiers for diagnosis and interpretation.

**Figure 4:**
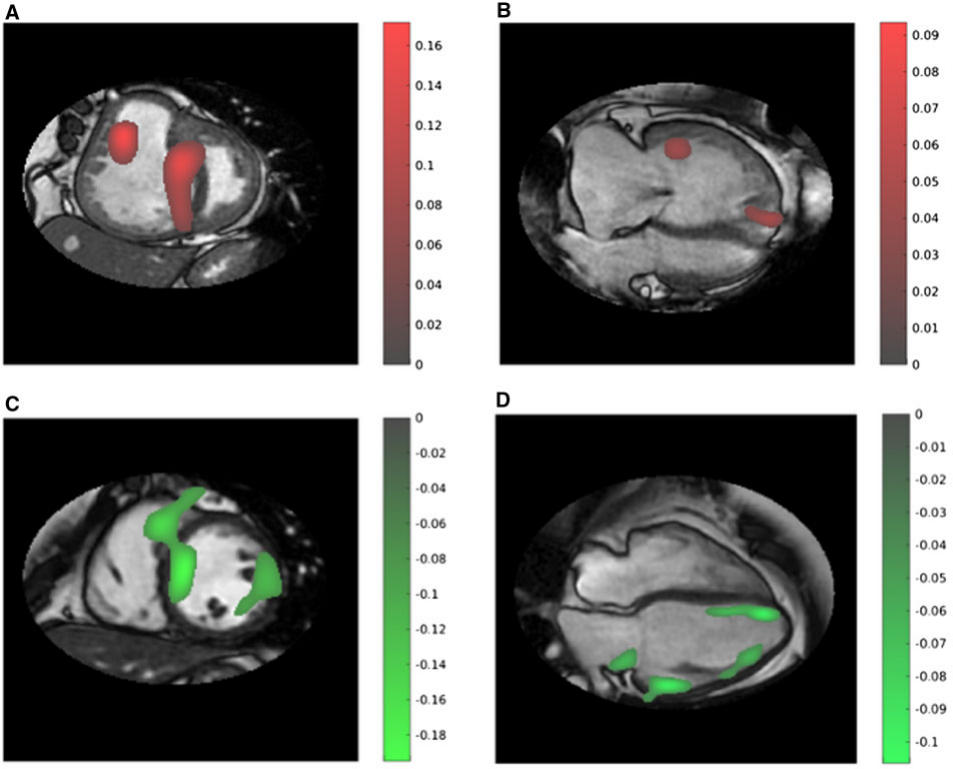
Representative feature map images: (i) short-axis (*A*) and four-chamber (*B*) CMR images in a patient with PAH, taken from Phase 11, early diastole. Notably, features are identified at the level of the interventricular septum and close to the right ventricular outflow tract within the blood pool. (ii) Short-axis (*C*) and four-chamber (*D*) CMR images in a patient without PAH, taken from Phase 1, end-diastole. Features are noted at the level of the mid chamber/apex septum and basal LV lateral wall. Red features are more indicative of PAH and green indicative of no PAH. The colour bars show the amplitude of the features detected, with a higher absolution value indicating a higher importance.

For short-axis imaging, features identified were most frequently located in the region of the interventricular septum *Figure 4A*. The learnt temporal features were most frequently identified and discriminative at end-systole. Four-chamber analysis also was highly diagnostic for PAH, where features associated with PAH (highlighted in red) were identified adjacent to the interventricular septum (*Figure 4B*).

New features were discovered on short-axis imaging, located at the interventricular septum at end-diastole/early systole which were indicative of no PH (highlighted in green) (*Figure 4C*). New features were also discovered on four-chamber images, green features are shown at the basal LV lateral wall at end-diastole indicating normality (*Figure 4D*), and red features are noted in the same location at end-systole indicating PAH.

### Diagnostic utility

#### PAH diagnosis

**Figure 5:**
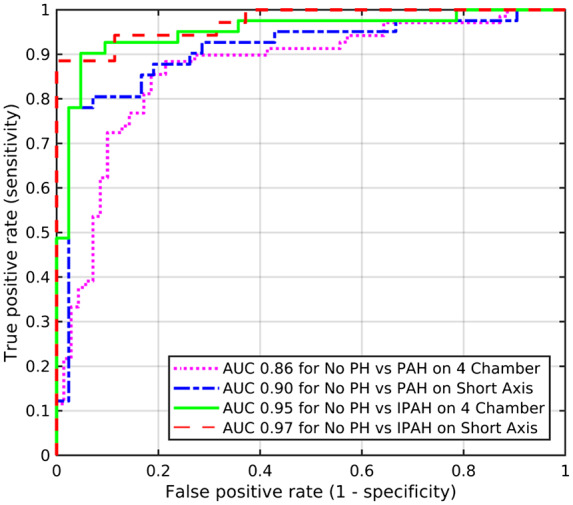
Receiver operating characteristic curve analysis showing diagnostic accuracy of the proposed machine learning approach in identification of PAH and IPAH, using short-axis (scale: 64 × 64) and four-chamber images (scale: 128 × 128) and small ellipse mask.

*Table 2* presents the ROC analysis results for established indices, as well as the proposed machine learning approach with scaling factor of 64 × 64 (7.50 mm) for short axis and 128 × 128 (3.75 mm) for four chambers, and with the best performing classifier (SVM/LR). The table also reports the respective confidence intervals. From 10-fold cross-validation of the machine learning approach, the average AUC for differentiation of patients with PAH from patients without PH was 0.90 for the short-axis cine images and 0.86 for the four-chamber cine images (*Figure 5*). This was achieved using the small elliptical mask for both short-axis and four-chamber images, rescaled to 64 × 64 and 128 × 128, respectively. *Table 3* shows diagnostic accuracy using different image scaling factors for short-axis and four-chamber cine images using the small ellipse mask. On short-axis and four-chamber imaging, the large elliptical mask and using no mask had weaker diagnostic accuracy (*Table 2*). SVM and LR both had good accuracy, with small differences only. For differentiation of PAH from without PAH, SVM had an AUC of 0.90, while LR had 0.87 for the short-axis cine images, and SVM had an AUC of 0.86, while LR had 0.78 for the four-chamber cine images. *Table 4* presents the sensitivity, specificity, PPV, and NPV of the machine learning approach.

**Table 2:** Receiver operating characteristic analysis results in AUC, with 95% confidence interval (CI) in parentheses for established indices and machine learning on short axis (scale: 64 × 64) and four chambers (scale: 128 × 128).

	PAH vs. no PH	IPAH vs. no PH
	AUC (95% CI)	AUC (95% CI)
Established indices		
RVEDVi	0.71 (0.64–0.78)	0.3 (0.77–0.90)
RVESVi	0.78 (0.72–0.84)	0.91 (0.86–0.96)
RVEF	0.79 (0.73–0.85)	0.89 (0.83–0.97)
RVSVi	0.54 (0.46–0.63)	0.54 (0.44–0.64)
VMi	0.84 (0.78–0.90)	0.92 (0.87–0.97)
Systolic septal angle	0.88 (0.84–0.93)	0.97 (0.94–0.99)
**Proposed machine learning approach**		
Short-axis: small ellipse	0.90 (0.85–0.93)	0.97 (0.89–1.0)
Short-axis: large ellipse	0.69 (0.62–0.76)	0.90 (0.83–0.97)
Short-axis: no mask	0.70 (0.61–0.79)	0.79 (0.70–0.88)
Four chambers: small ellipse	0.86 (0.80–0.92)	0.95 (0.89–1.0)
Four chambers: large ellipse	0.77 (0.72–0.81)	0.85 (0.78–0.92)
Four chambers: no mask	0.69 (0.63–0.75)	0.79 (0.73–0.85)

RVEDVi, right ventricular end-diastolic volume index; RVEF, right ventricular ejection fraction; RVESVi, right ventricular end-systolic volume index; RVSVi, right ventricular stroke volume index; VMI, ventricular mass index.

**Table 3:** Comparison of diagnostic accuracy using different image scaling factors for short-axis (SA) and four-chamber cine images using the small ellipse mask

		PAH vs. no PH		IPAH vs. no PH	
		SA	Four chambers	SA	Four chambers
Scaling factor	Resolution (mm)	AUC	AUC	AUC	AUC
32 × 32	15.0	0.92	0.84	0.94	0.91
64 × 64	7.50	0.90	0.85	0.97	0.94
128 × 128	3.75	0.89	0.86	0.96	0.95
256 × 256	1.89	0.88	0.87	0.94	0.95
512 × 512 (original scale)	0.94	0.86	0.87	0.95	0.91

**Table 4:** Sensitivity, specificity, positive predictive value (PPV), and negative predictive value (NPV) of the proposed machine learning approach in identification of PAH and IPAH, using short-axis (scale: 64 × 64) and four-chamber images (scale: 128 × 128).

	Sensitivity	Specificity	PPV	NPV	Accuracy
No PH vs. PAH					
Short axis	0.89	0.81	0.91	0.78	0.87
Four chambers	0.86	0.81	0.91	0.73	0.85
No PH vs. IPAH					
Short axis	0.96	0.87	0.89	0.95	0.92
Four chambers	0.93	0.90	0.90	0.93	0.91

Among existing CMR diagnostic measurements for the diagnosis of PAH, ventricular mass index, and interventricular septal angle were the top predictive manually drawn measurements, with similar high diagnostic utility of AUC = 0.84 and 0.88, respectively.

#### IPAH diagnosis

The AUC values for the machine learning approach were higher for differentiation of no PH from IPAH, 0.97 for the short-axis cine images and 0.95 for the four-chamber cine images (*Figure 5*). This was again achieved using the small elliptical mask. On short-axis and four-chamber imaging, the large elliptical mask and using no mask had weaker diagnostic accuracy (*Table 2*). For differentiation of no PH from IPAH, SVM had an AUC of 0.97, while LR had 0.95 for the short-axis cine images, and both SVM and LR had an AUC of 0.95 for the four-chamber cine images. *Table 4* presents the sensitivity, specificity, PPV, and NPV of the machine learning approach.

The results from existing manually drawn CMR diagnostic measurements were similarly high, where ventricular mass index and septal angle have AUC of 0.92 and 0.97, respectively.

### Preprocessing results

Registration was successful in all cases as judged by visual inspection. Error distance measured in unit pixel (in an image of 512 × 512) was 7.1 (SD 3.7, range 0.32–20.1) for four-chamber images and 4.1 (SD 2.3, range 0.3–10.6) for short axis. The small elliptical mask consistently gave the best classification results for both short-axis and four-chamber imaging (outperforming large mask and no mask).

## Discussion

This study shows for the first time that a machine learning workflow based on multilinear subspace learning applied to CMR cine images can provide a rapid diagnostic assessment without manual segmentation of images and can differentiate patients with and without PAH with high accuracy. The diagnostic utility of the machine learnt image and temporal features was marginally higher than that of time-consuming, manually drawn diagnostic CMR measurements used in PAH, with comparable variations in confidence intervals. However, the proposed machine learning workflow has much lower cost and much less variability than the manual segmentation-based indices produced by PH imaging experts at a specialist centre. Learnt features were visualized in feature maps corresponding to cardiac phases, confirming known and also identifying potentially new diagnostic features in PAH.

Identified features were most frequently located in the region of the interventricular septum for short-axis imaging. We postulate that ‘the diagnostic value of interventricular septal deviation has been learnt automatically by the machine’. We know that septal deviation is a feature with added diagnostic value, particularly when measured at end-systole.^9^^,^^25^ In keeping with higher accuracy of septal position at end-systole, the learnt temporal features were most frequently identified at end-systole as providing the highest discriminative power. New features were discovered on short-axis imaging, e.g. green features located at the interventricular septum at end-diastole/early systole are indicative of normality (*Figure 4C*).

Four-chamber analysis also was highly diagnostic for PAH, with red features identified adjacent to the interventricular septum as expected, *Figure 4B* but also in the RV free wall, particularly the tricuspid annulus at end-diastole likely relating to abnormal free wall contraction. New features were also discovered on four-chamber images. Green features were shown at the basal LV lateral wall at end-diastole indicating normality (*Figure 4C and D*), and PAH features were noted in the same location at end-systole indicating PAH. These features may reflect impact of PAH on LV function.

Blood pool disease features in the right ventricle were identified. RV blood pool features indicating PAH diagnosis (red, *Figure 4A and B*) have been found during systole and those indicating normality (green, *Figure 4D*) have been found during diastole, likely relating to systolic and diastolic RV function. Evaluation of flow sequences using the proposed machine learning workflow is a next step to better understand the diagnostic value of intracardiac flow patterns.

A major advantage of the proposed tensor-based machine learning approach is the removal of the requirement for manual segmentation. Typically regions of interest, lines, or angles are drawn by experienced observers directly on the images to derive cardiac and pulmonary measurements. Such measurements are affected by interobserver variability even in expert hands, particularly for RV measurements.^19^ Furthermore, it is known that the reproducibility of RV measurements is inferior to those of the left ventricle. The manual landmarking of only three points in our machine learning approach is easier and faster to perform (a few seconds) and would require little training to undertake.

Automatic detection and segmentation of the ventricles using machine learning is also showing promise.^26^ The data derived from these approaches may reduce the need for manual adjustments that are currently labour intensive, especially for the right ventricle. Such approaches are however limited by the accuracy of manual segmentation used to train the algorithms. The approach proposed in the present paper has the great advantage of assessing all pixels within a mask in each image of a sequence and has the potential to pick up diagnostic features from within or outside of the ventricles, e.g. atrial size and function, and pericardial and pleural effusions. This allows the computer to identify discriminative features in an unbiased fashion. A small number of diagnostic features were identified outside of the heart (e.g. *Figure 4C*), which indicates the need of further work to investigate their pathophysiological significance and potential diagnosis value. Other applications include treatment response assessment, subtyping, severeness assessment, and pressure/PVR prediction. In particular, the range of prognostic features from the right and left ventricles,^4^^,^^27–29^ the atria,^30^ and features of decompensation such as pericardial effusion^31^ may be identified using the proposed approach.

It is known that PH is underdiagnosed and machine learning may help to improve diagnostic rates and ultimately improve outcomes.^32^^,^^33^ A prior study using machine learning to measure RV tissue motion has shown good prognostic value in PH,^26^ and association with metabolic profiles.^34^ In the development of the proposed machine learning approach, we aim to gain insights into the features identified for further analyses (e.g. biomarker identification/treatment). Therefore, we favour relatively simple, yet interpretable machine learning methods, such as linear methods, over highly complex, but opaque black-box methods, such as the popular deep neural networks.^35^ In particular, linear methods allow direct visualization of the importance of individual features to assist interpretation.^21^

The length of time to manually analyse a CMR study is typically 10–15 min, depending on the complexity of the analysis. There can be variability between observers in their approach to the analysis and there are also subtle differences in the software provided by vendors. The machine learning approach proposed here, if used to test an individual short-axis sequence, would take less than 10 s, including manual landmarking (dominating the time cost), and automated preprocessing and diagnostic output. The automated process after landmarking would take less than 1 s.

Masks of different sizes were studied with best results obtained using smaller more focused elliptical masks around the heart. This finding was expected given the effect of PH on the heart and the characteristic imaging features of high pulmonary arterial pressure. For both IPAH and PAH diagnosis, high accuracy was found even when short-axis images were rescaled to 64 × 64 and four-chamber images were rescaled to 128 × 128, compared with the original scale 512 × 512 (see *Table 3*). At these lower resolutions, the structural information may play a smaller role and it is the functional information that is driving the differentiation of health from disease, likely lower RV function, abnormal RV and LV flow patterns, and abnormal septal motion. Studying larger cohorts may allow higher accuracy with input of high-quality data.

### Limitations

Landmarks were manually drawn to register the patient sequences to one another. Fully automated registration of sequences, e.g. via automated landmark detection using deep learning, is an area for future work that can further reduce manual user input to just a visual check to ensure accurate landmark detection and make corrections if necessary for quality assurance.

Whilst typical interobserver variability is removed using our automatic machine learning method, quantification of the impact of scan-to-scan variability is an area for future research. Only four-chamber images and mid-chamber short-axis cine images were used for this study. Further work includes taking as input multislice cardiac volumetric data and 4D flow data, in addition to flow imaging, which may improve diagnostic accuracy. Further works also include testing this approach in unselected patients with suspected PH and validating the approach in a second cohort, in addition to validation in a multivendor multicentre setting. In addition, trialling such an approach in other challenging diagnostic and prognostic cardiac scenarios would be of value. Factoring image quality score into the approach is also an important area for further research to explore the potential of machine learning in low-quality cases that are particularly challenging for humans.

## Conclusion

A tensor-based machine learning approach has been developed to analyse CMR images without manual image segmentation. It has holistically identified predictive and interpretable temporal and image features and allows rapid and accurate diagnosis of PAH.

## Funding

This work was supported by Wellcome (215799/Z/19/Z and 205188/Z/16/Z), EPSRC (EP/R014507/1), NIHR (NIHR-RP-R3-12-027), and MRC (MR/M008894/1). The views expressed in this publication are those of the author(s) and not necessarily those of the NHS, the National Institute for Health Research or the Department of Health. D. Capener was partly funded by an unrestricted research grant from Bayer.

**Conflict of interest:** none declared.

## References

[1] Kiely DG, Elliot CA, Sabroe I, Condliffe R. Pulmonary hypertension: diagnosis and management. BMJ 2013;346:f2028. 23592451 10.1136/bmj.f2028

[2] Swift AJ, Rajaram S, Condliffe R, Capener D, Hurdman J, Elliot C et al Pulmonary artery relative area change detects mild elevations in pulmonary vascular resistance and predicts adverse outcome in pulmonary hypertension. Invest Radiol 2012;47:571–7. 22914228 10.1097/RLI.0b013e31826c4341

[3] Schäfer M, Myers C, Brown RD, Frid MG, Tan W, Hunter K et al Pulmonary arterial stiffness: toward a new paradigm in pulmonary arterial hypertension pathophysiology and assessment. Curr Hypertens Rep 2016;18:4. 26733189 10.1007/s11906-015-0609-2

[4] Gan C-J, Lankhaar J-W, Westerhof N, Marcus JT, Becker A, Twisk JWR et al Noninvasively assessed pulmonary artery stiffness predicts mortality in pulmonary arterial hypertension. Chest 2007;132:1906–12. 17989161 10.1378/chest.07-1246

[5] Friesen RM, Schäfer M, Ivy DD, Abman SH, Stenmark K, Browne LP et al Proximal pulmonary vascular stiffness as a prognostic factor in children with pulmonary arterial hypertension. Eur Heart J Cardiovasc Imaging 2019;20:209–17. 29788051 10.1093/ehjci/jey069 PMC6343079

[6] Saba TS, Foster J, Cockburn M, Cowan M, Peacock AJ. Ventricular mass index using magnetic resonance imaging accurately estimates pulmonary artery pressure. Eur Respir J 2002;20:1519–24. 12503713 10.1183/09031936.02.00014602

[7] Moceri P, Bouvier P, Baudouy D, Dimopoulos K, Cerboni P, Wort SJ et al Cardiac remodelling amongst adults with various aetiologies of pulmonary arterial hypertension including Eisenmenger syndrome—implications on survival and the role of right ventricular transverse strain. Eur Heart J Cardiovasc Imaging 2017;18:1262–70. 28011668 10.1093/ehjci/jew277

[8] Hulshof HG, Eijsvogels TMH, Kleinnibbelink G, Dijk A. V, George KP, Oxborough DL et al Prognostic value of right ventricular longitudinal strain in patients with pulmonary hypertension: a systematic review and meta-analysis. Eur Heart J Cardiovasc Imaging 2019;20:475–84. 30169841 10.1093/ehjci/jey120

[9] Dellegrottaglie S, Sanz J, Poon M, Viles-Gonzalez JF, Sulica R, Goyenechea M et al Pulmonary hypertension: accuracy of detection with left ventricular septal-to-free wall curvature ratio measured at cardiac MR. Radiology 2007;243:63–9. 17392248 10.1148/radiol.2431060067

[10] Swift AJ, Rajaram S, Hurdman J, Hill C, Davies C, Sproson TW et al Noninvasive estimation of PA pressure, flow, and resistance with CMR imaging. JACC Cardiovasc Imaging 2013;6:1036–47. 23769494 10.1016/j.jcmg.2013.01.013

[11] Sato T, Tsujino I, Ohira H, Oyama-Manabe N, Ito YM, Yamada A et al Right atrial volume and reservoir function are novel independent predictors of clinical worsening in patients with pulmonary hypertension. J Heart Lung Transplant 2015;34:414–23. 25813768 10.1016/j.healun.2015.01.984

[12] Crawley SF, Johnson MK, Dargie HJ, Peacock AJ. LA volume by CMR distinguishes idiopathic from pulmonary hypertension due to HFpEF. JACC Cardiovasc Imaging 2013;6:1120–1. 24135327 10.1016/j.jcmg.2013.05.014

[13] Currie BJ, Johns C, Chin M, Charalampopolous T, Elliot CA, Garg P et al CT derived left atrial size identifies left heart disease in suspected pulmonary hypertension: derivation and validation of predictive thresholds. Int J Cardiol 2018;260:172–7. 29530618 10.1016/j.ijcard.2018.02.114 PMC5899969

[14] Rajaram S, Swift AJ, Condliffe R, Johns C, Elliot CA, Hill C et al CT features of pulmonary arterial hypertension and its major subtypes: a systematic CT evaluation of 292 patients from the ASPIRE Registry. Thorax 2015;70:382–7. 25523307 10.1136/thoraxjnl-2014-206088 PMC4392204

[15] Park B, Dittrich HC, Polikar R, Olson L, Nicod P. Echocardiographic evidence of pericardial effusion in severe chronic pulmonary hypertension. Am J Cardiol 1989;63:143–5. 2909152 10.1016/0002-9149(89)91105-3

[16] Janda S, Shahidi N, Gin K, Swiston J. Diagnostic accuracy of echocardiography for pulmonary hypertension: a systematic review and meta-analysis. Chest 2010;138:923A. 10.1136/hrt.2010.212084 21357375

[17] Taleb M, Khuder S, Tinkel J, Khouri SJ. The diagnostic accuracy of Doppler echocardiography in assessment of pulmonary artery systolic pressure: a meta-analysis. Echocardiography 2013;30:258–65. 23227919 10.1111/echo.12061

[18] Puchalski MD, Williams RV, Askovich B, Minich LL, Mart C, Tani LY. Assessment of right ventricular size and function: echo versus magnetic resonance imaging. Congenit Heart Dis 2007;2:27–31. 18377513 10.1111/j.1747-0803.2007.00068.x

[19] Grothues F, Moon JC, Bellenger NG, Smith GS, Klein HU, Pennell DJ. Interstudy reproducibility of right ventricular volumes, function, and mass with cardiovascular magnetic resonance. Am Heart J 2004;147:218–23. 14760316 10.1016/j.ahj.2003.10.005

[20] Lu H, Plataniotis KN, Venetsanopoulos A. Multilinear Subspace Learning: Dimensionality Reduction of Multidimensional Data. CRC Press; 2013, p. 296.

[21] Song X, Meng L, Shi Q, Lu H. Learning tensor-based features for whole-brain fMRI Classification. In: *International Conference on Medical Image Computing and Computer Assisted Intervention, Munich, Germany* 2015 p. 613–620.

[22] Lu H, Plataniotis KNK, Venetsanopoulos AN. MPCA: multilinear principal component analysis of tensor objects. IEEE Trans Neural Netw 2008;19:18–39. 18269936 10.1109/TNN.2007.901277

[23] Katz J, Whang J, Boxt LM, Barst RJ. Estimation of right ventricular mass in normal subjects and in patients with primary pulmonary hypertension by nuclear magnetic resonance imaging. J Am Coll Cardiol 1993;21:1475–81. 8473659 10.1016/0735-1097(93)90327-w

[24] Swift AJ, Rajaram S, Condliffe R, Capener D, Hurdman J, Elliot CA et al Diagnostic accuracy of cardiovascular magnetic resonance imaging of right ventricular morphology and function in the assessment of suspected pulmonary hypertension results from the ASPIRE registry. J Cardiovasc Magn Reson 2012;14:40. 22720870 10.1186/1532-429X-14-40 PMC3419131

[25] Johns CS, Kiely DG, Rajaram S, Hill C, Thomas S, Karunasaagarar K et al Diagnosis of pulmonary hypertension with cardiac MRI: derivation and validation of regression models. Radiology 2019;290:61–8. 30351254 10.1148/radiol.2018180603 PMC6314564

[26] Dawes TJW, Marvao A. D, Shi W, Fletcher T, Watson GMJ, Wharton J et al Machine learning of three-dimensional right ventricular motion enables outcome prediction in pulmonary hypertension: a cardiac MR imaging study. Radiology 2017;283:381–90. 28092203 10.1148/radiol.2016161315 PMC5398374

[27] Swift AJ, Capener D, Johns C, Hamilton N, Rothman A, Elliot C et al Magnetic resonance imaging in the prognostic evaluation of patients with pulmonary arterial hypertension. Am J Respir Crit Care Med 2017;196:228–39. 28328237 10.1164/rccm.201611-2365OC PMC5519970

[28] Mc van de V, Kind T, Marcus JT, Mauritz G-J, Heymans MW, Bogaard H-J et al Progressive right ventricular dysfunction in patients with pulmonary arterial hypertension responding to therapy. J Am Coll Cardiol 2011;58:2511–9. 22133851 10.1016/j.jacc.2011.06.068

[29] Lewis RA, Johns CS, Cogliano M, Capener D, Tubman E, Elliot CA et al Identification of cardiac MRI thresholds for risk stratification in pulmonary arterial hypertension. Am J Respir Crit Care Med 2019. doi: 10.1164/rccm.201909-1771OC. [Epub ahead of print]. 10.1164/rccm.201909-1771OC PMC7049935 31647310

[30] Darsaklis K, Dickson ME, Cornwell W 3rd, Ayers CR, Torres F, Chin KM et al Right atrial emptying fraction non-invasively predicts mortality in pulmonary hypertension. Int J Cardiovasc Imaging 2016;32:1121–30. 27076226 10.1007/s10554-016-0883-3

[31] Batal O, Dardari Z, Costabile C, Gorcsan J, Arena VC, Mathier MA. Prognostic value of pericardial effusion on serial echocardiograms in pulmonary arterial hypertension. Echocardiography 2015;32:1471–6. 25682779 10.1111/echo.12909

[32] Gall H, Hoeper MM, Richter MJ, Cacheris W, Hinzmann B, Mayer E. An epidemiological analysis of the burden of chronic thromboembolic pulmonary hypertension in the USA, Europe and Japan. Eur Respir Rev 2017;26:160121. 28356407 10.1183/16000617.0121-2016 PMC9488926

[33] Bergemann R, Allsopp J, Jenner H, Daniels FA, Drage E, Samyshkin Y et al; SPHInX Project team . High levels of healthcare utilization prior to diagnosis in idiopathic pulmonary arterial hypertension support the feasibility of an early diagnosis algorithm: the SPHInX project. Pulm Circ 2018;8:204589401879861. 10.1177/2045894018798613 PMC6311599 30187824

[34] Attard MI, Dawes TJW, Marvao A. D, Biffi C, Shi W, Wharton J et al Metabolic pathways associated with right ventricular adaptation to pulmonary hypertension: 3D analysis of cardiac magnetic resonance imaging. Eur Heart J Cardiovasc Imaging 2019;20:668–76. 30535300 10.1093/ehjci/jey175 PMC6529902

[35] Loh BCS, Then P. Deep learning for cardiac computer-aided diagnosis: benefits, issues & solutions. mHealth 2017;3:45. 29184897 10.21037/mhealth.2017.09.01 PMC5682365

